# The complete mitochondrial genome sequence of the greater wax moth *Galleria mellonella* (Insecta, Lepidoptera, Pyralidae): sequence and phylogenetic analysis comparison based on whole mitogenome

**DOI:** 10.1080/23802359.2017.1390418

**Published:** 2017-10-17

**Authors:** Yeong-Jun Park, Chang Eon Park, Sung-Jun Hong, Byung Kwon Jung, Jerald Conrad Ibal, Gun-Seok Park, Jae-Ho Shin

**Affiliations:** a School of Applied Biosciences, Kyungpook National University, Daegu, Republic of Korea;; b Department of Biomedical Engineering, The University of Texas at Austin, Austin, TX, USA;; c Institute of Agricultural Science & Technology, Kyungpook National University, Daegu, Republic of Korea

**Keywords:** Galleria mellonella, honeycomb moth, greater wax moth

## Abstract

The larva of *Galleria mellonella* is widely used as a model organism for *in vivo* toxicology and pathogenicity testing. Here, we report complete sequence of the mitochondrial genome (mitogenome) from *G. mellonella*, which is comprised of 15,320 base pairs encoding 13 protein-coding genes, two ribosomal RNAs, 22 transfer RNAs, and an A + T rich region. The overall base composition was G + C: 19.6%, A + T: 80.4%, with an apparent AT bias. Phylogenetic analysis using whole mitogenome revealed that *G. mellonella* was closely related to *Corcyra cephalonica*, which is in the same Pyralidae family.

The honeycomb moth or greater was moth *Galleria mellonella* belongs to the family Pyralidae, and is the only member of the genus. The moth spread throughout the world, including Asia, Europe and North America. In the beekeeping industry, the *G. mellonella* is known as the serious insect of bees and since it transports sacbrood virus which causes honey bee disease (Al-Ghamdi 1990). Moreover, waxworms of this moth is known to be an excellent model organism for bacterial or fungal pathogenicity testing (Mylonakis et al. 2005; Peleg et al. 2009; Fuchs et al. 2010; Mukherjee et al. 2010). Also, the larvae of this organism are models for studying the innate immune system (Mylonakis et al. 2005; Mukherjee et al. 2010). Despite of this importance of the organism, there is no existing genomic research.

A sample of *G. mellonella* was collected in Gunwi-Gun, Gyeongsangbuk-Do, Republic of Korea (GIS: 36° 7′ 5.3″N 128°39′19.0″E). The whole-body specimen is being kept under the voucher number NIBRGR0000375858 in National Institute of Biological Resources, Republic of Korea. The mitochondrial DNA was amplified using the modified mitochondrial specific primer sets (Simon et al. 1994). A PCR product that was not well amplified was judged to contain A + T rich region (Su et al. 1996). The fragment was successfully amplified after decreasing extension temperature in the PCR cycle. NGS library preparation and sequencing were performed using the Ion Torrent PGM platform (Life Technologies/Thermo Fisher Scientific, Waltham, MA). CLC genomics workbench 7.5 (CLC Bio, Denmark) was used for the mitochondrial DNA assembly and annotation. PCGs and tRNAs in the mitogenome were confirmed by NCBI Basic Local Alignment Search Tool (BLAST) (Altschul et al. 1990) and tRNAscan-SE 1.21 (Lowe and Eddy 1997), respectively. Phylogenetic analysis was performed using whole mitogenome in the Lepidoptera order.

The complete mitogenome sequence of *G. mellonella* was submitted to GenBank (accession number: NC028532). The mitogenome was composed of circular DNA with 15,320 bp. The overall nucleotide composition was asymmetric (A: 38.6%, T: 41.8%, G: 7.5%, C: 12.1%,) with an apparent AT bias. It contains 13 PCGs, 2 rRNA genes, 22 tRNA genes, and a D-loop with four kinds of start codons and two kinds of stop codons. Phylogenetic relationship of *G. mellonella* was demonstrated based on whole mitogenome sequence from current and published study. In analysis, sequence alignment, bootstrap method with 1,000 replication value, and phylogenetic tree illustration were performed using MEGA 6.0 (Tamura et al. 2013). Phylogenetic trees with whole mitogenome sequence was show that *G. mellonella* was most closely related to *C. cephalonica* located in Pyralidae family (Figure 1). Based on these results, mitogenome of *G. mellonella* can contribute to the phylogenetic knowledge of the genus *Galleria*.

**Figure 1.**
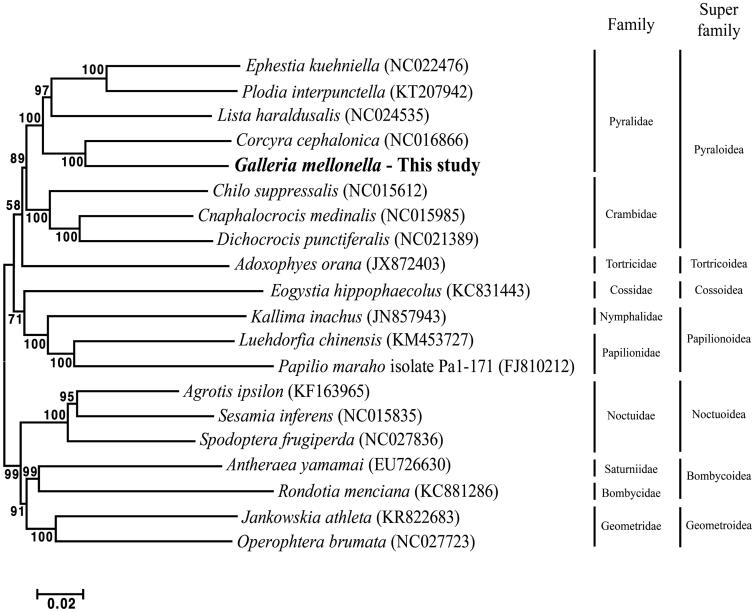
Phylogenetic tree based on 20 whole mitogenomes constructed using maximum likelihood approach. The number in the phylogenetic tree is bootstrap probability value and presented in the above branches. On the right side, the stick means range which includes certain family or super family. The GenBank accession numbers are indicated after the scientific name.
